# A Facile Self-assembly Synthesis of Hexagonal ZnO Nanosheet Films and Their Photoelectrochemical Properties

**DOI:** 10.1007/s40820-015-0068-y

**Published:** 2015-10-15

**Authors:** Bin Zhang, Faze Wang, Changqing Zhu, Qiang Li, Jingnan Song, Maojun Zheng, Li Ma, Wenzhong Shen

**Affiliations:** 1 Key Laboratory of Artificial Structures and Quantum Control (Ministry of Education), Department of Physics and Astronomy, Shanghai Jiao Tong University, Shanghai, 200240 People’s Republic of China; 2 Collaborative Innovation Center of Advanced Microstructures, Nanjing University, Nanjing, 210093 People’s Republic of China; 3 School of Chemistry & Chemical Technology, Shanghai Jiao Tong University, Shanghai, 200240 People’s Republic of China

**Keywords:** Zinc oxide, Nanosheet film, Self-assemble, Galvanic displacement method, Photoelectrochemical property

## Abstract

Here, large-scale and uniform hexagonal zinc oxide (ZnO) nanosheet films were deposited onto indium tin oxide (ITO)-coated transparent conducting glass substrates via a facile galvanic displacement deposition process. Compared with other commonly used solution methods, this process avoids high temperature and electric power as well as supporting agents to make it simple and cost-effective. The as-fabricated ZnO nanosheet films have uniform hexagonal wurtzite structure. The photoelectrochemical (PEC) cell based on ZnO nanosheet film/ITO photoelectrode was also fabricated and its performance was improved by optimizing the solution concentration. A higher photocurrent density of ~500 μA cm^−2^ under AM 1.5 G simulated illumination of 100 mW cm^−2^ with zero bias potential (vs. Ag/AgCl electrode) was obtained, which may ascribe to the increased surface-to-volume ratio of disordered ZnO nanosheet arrays. Our developed method may be used to deposit other oxide semiconductors, and the ZnO nanosheet film/ITO PEC cell can be used to design low-cost optoelectronic and photoelectrochemical devices.

## Introduction

Over the past decades, there has been an increasing scientific interest in oxide semiconductors (such as TiO_2_, ZnO, Fe_2_O_3_, CuO, NiO, and so on) [1–3], because of their numerous potential technological applications, including photovoltaic device [4], lithium ion battery [5], photocatalysis, and optoelectronic device [6, 7]. Among the oxide semiconductors, ZnO as a prototypical *n*-type conducting oxide has attracted considerable attentions for wide usages in piezoelectric device, ultraviolet optoelectronics detectors [8, 9], low-cost dye-sensitized solar cells [10], gas sensors [11, 12], photocatalysis, and photoelectrochemical (PEC) devices [13, 14]. Most of these applications are based on the advantages of its abundance, low cost, non-toxicity, chemical stability and the possibility of growing ordered nanostructures, and strong exciton binding energy [15]. Especially, nanostructured ZnO exhibits enhanced performance and provided an ideal system to study the influence of surface effects and interface science on photoelectrochemical properties due to their large surface-to-volume ratios [16–18].

To date, ZnO and other nanostructured materials have been assembled and studied by various methods and means [19–22], including magnetron sputtering [23], chemical vapor deposition [24], hydrothermal process [25], electrochemical deposition [26–28], electroless deposition [29, 30], and other combination of methods [31]. Among all these techniques, electroless deposition presents several advantages such as low cost, large-scale deposition, and low-temperature processing. Here, we explored a facile solution-based galvanic displacement deposition technique to self-assembly synthesis uniform hexagonal ZnO nanosheet film on ITO glass substrate under ambient conditions. ZnO nanosheet film/ITO PEC cell and its photoelectrochemical property were also investigated.

## Experimental Details

### Synthesis of the ZnO Nanosheet Films

Compared with other common electrodeposition methods, an electroless deposition process was employed to fabricate ZnO nanosheets. A typical galvanic cell system with two half-cells (A and B) was used to generate a current by coupling oxidation and reduction reactions in a spontaneous process, in which the A cell solution is 5–15 mM ZnSO_4_ and the B cell solution is 0.25 M NaOH. The two half-cells were connected by a porous salt bridge that supplied ions to maintain charge neutrality during current flow. The galvanic cell deposition system is composed of Al sheet (99.99 % purity) and ITO conducting glass (sheet resistance of 10 Ω cm^−2^), which acted as anode and cathode, respectively. The two electrodes were short-circuited externally through a metal copper wire. Before deposition, 4 cm × 2 cm Al sheet and ITO glass were, respectively, cleaned in acetone, ethanol, and deionized water for 5 min. Then, the Al sheet was immerged into A cell solution and the ITO glass was immerged into B cell solution without stirring and oxygen gas bubbling at room temperature. In order to obtain higher crystal quality, the as-prepared samples were annealed at 550 °C for 60 min with the increasing rate of 10 °C min^−1^ in an air atmosphere.

### Characterization

The surface morphology of the as-deposited ZnO nanosheet films was obtained by field emission scanning electron microscopy (FE-SEM; FEI Sirion 200, Holland). A D8 ADVANCE DA VINCI X-ray diffractometer (XRD, Bruker, German) was employed to verify the crystal structure of the samples using Cu *K*α radiation (*λ* = 0.15418 nm) with a scanning rate of 5° min^−1^. The photoluminescence (PL) spectrum of the as-resulting ZnO films was obtained using the Jobin–Yvon LabRam HR 800 UV system with a 325-nm laser at room temperature. The UV–Vis spectra of the ZnO samples were obtained through UV–Vis spectrophotometer (PerkinElmer Lambda 950, America).

### PEC Cell Preparation and PEC Characterization

PEC cell was fabricated by placing a copper wire onto a bare portion of the ITO conducting substrate and securing with high-purity silver conducting glue. Then, the part active area of ZnO nanosheet films was sealed with epoxy resin leaving an exposed working electrode surface area of 1 cm × 1 cm. An electrochemical workstation (Princeton Applied Research, PARSTAT 4000, America) was used to study the photoelectrochemical property of the samples. The photoelectrochemical experiment was performed in a conventional three electrode, in which the resulting ZnO nanosheet film/ITO substrate acted as working electrode (illuminating area of 1 cm^2^), a platinum net (surface area of 1 cm^2^) as counter electrode, and an Ag/AgCl as reference electrode. The PEC experiments were carried out in a mixture solution of 0.35 M Na_2_S and 0.25 M Na_2_SO_3_ under AM 1.5 G (100 mW cm^−2^) simulated illumination, which was provided by a 300 W xenon lamp (Beijing Perfectlight Technology, PLS-SXE300C, China) equipped with an AM 1.5 filter. The illumination intensity was measured with a solar simulator spectroradiometer (EKO instrument, LS-100, Japan).

## Results and Discussion

**Fig. 1:**
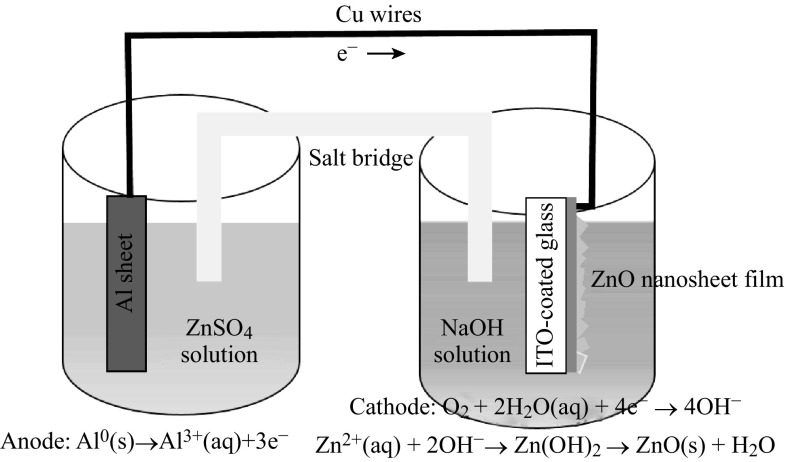
The schematic drawing of the experimental setup used for the fabrication of hexagonal ZnO nanosheet films

The schematic drawing of the experimental setup used for the fabrication ZnO nanosheet films is shown in Fig. 1. When the Al electrode, connected with the ITO glass externally, was dipped into the NaOH solution, Al^3+^ ions formed in the solution due to the dissolution of Al foil. Then, the released electrons moved through the externally short-circuited path to the ITO electrode. At the same time, the electron prompted the basic electrochemical reduction of oxygen (O_2_) in the aqueous solution, and then led to the formation of OH^−^ ions on ITO substrate surface. Finally, the Zn^2+^ ions in the solution were attracted by the corresponding OH^−^ ions on the ITO surface, and an intermediate Zn(OH)_2_ was formed and rapidly converted to ZnO. The growth mechanism of ZnO nanosheet film has similar electrochemical reaction with the electrodeposition [32]. The reactions may be as follows:

$${\text{Anode:}}{\text{ Al}}^{0} \left( {\text{s}} \right) \to {\text{Al}}^{ 3+ } \left( {\text{aq}} \right) + 3 {\text{e}}^{ - }$$

$${\text{Cathode:}}{\text{ O}}_{ 2} + 2 {\text{H}}_{ 2} {\text{O }}\left( {\text{aq}} \right) + 4 {\text{e}}^{ - } \to 4 {\text{OH}}^{ - }$$

$${\text{Zn}}^{ 2+ } \left( {\text{aq}} \right) + 2 {\text{OH}}^{ - } \to {\text{Zn}}\left( {\text{OH}} \right)_{ 2} \to {\text{ZnO }}\left( {\text{s}} \right) + {\text{H}}_{ 2} {\text{O}}.$$

In the process of nanosheet film formation, ZnO nanocrystals were primarily generated and then they were self-assembled into ordered hexagon nanostructure. These staggered arrangement nanosheet formed ZnO film in the substrate surface finally. Actually, semiconductor self-assembly is a complex phenomenon that depends on the interplay of several physical factors and competing interactions of different nature. A thorough understanding of the self-assembly mechanism of nanocrystals to organize into ordered nanostructure is one of the keys of future nanoscience [33].

**Fig. 2:**
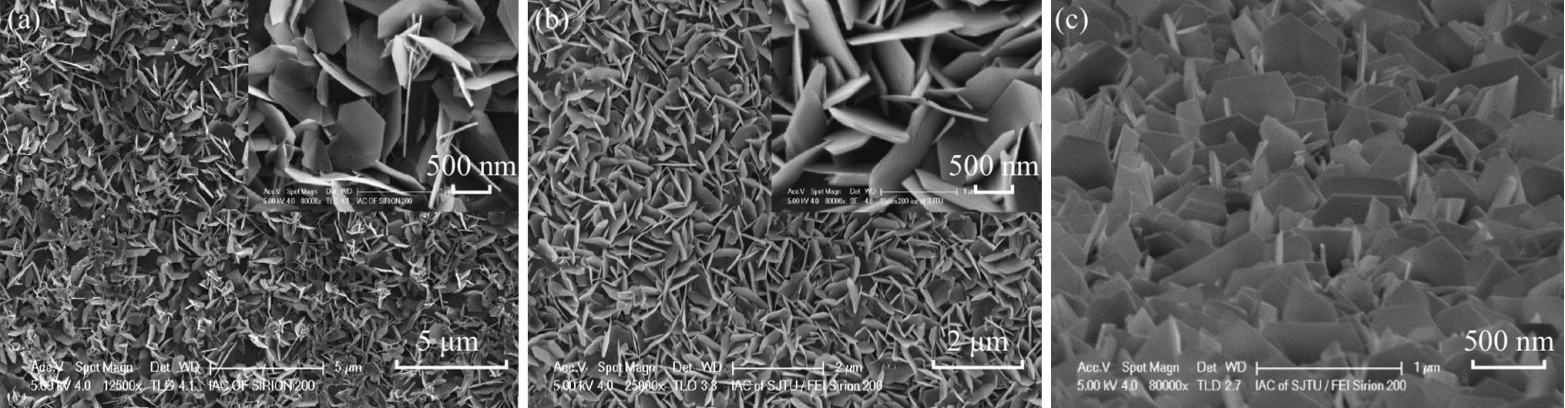
FE-SEM images of the as-prepared hexagonal ZnO nanosheet films grown on the ITO-coated glass substrate with different concentrations of ZnSO_4_. **a** 5 mM and **b** 10 mM, *top view*. **c** 10 mM, *side view*

Figure 2 shows FE-SEM images of the top and side view of the hexagonal ZnO nanosheet films grown on the ITO substrate at 5 and 10 mM ZnSO_4_ aqueous solution for 2 h, and annealed at 550 °C for 1 h. One can see that the ZnO had quite perfect hexagon and large-scale irregular arrangement. The samples shown in Fig. 2a, b were, respectively, grown at 5 mM ZnSO_4_ solution and 10 mM ZnSO_4_. It can be seen that the concentration of ZnSO_4_ aqueous solution plays an important role in the size and thickness of hexagonal ZnO nanosheets, as well as the surface-to-volume ratios.

**Fig. 3:**
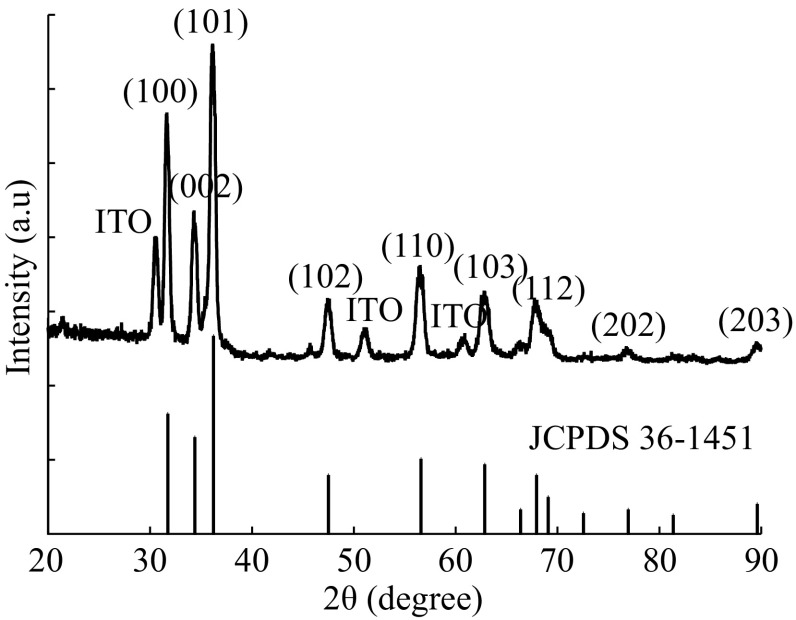
XRD pattern of the as-annealed hexagonal ZnO nanosheet film at 550 °C in open air conditions

Figure 3 shows XRD pattern of the hexagonal ZnO nanosheet films on ITO by electroless depositing at 7.5 mM ZnSO_4_ for 2 h under room temperature and post-annealing at 550 °C for 1 h in open air conditions. All diffraction peaks correspond to the standard diffraction of a hexagonal wurtzite ZnO crystal (JCPDS 36-1451) [34]. The major diffraction peaks have sharp features, corresponding to the (100), (002), (101), (102), (110), (103), and (112) planes, which is due to the disorder arrangement of ZnO nanosheets on the ITO. At the same time, the peaks from the ITO conducting substrates were observed as well.

**Fig. 4:**
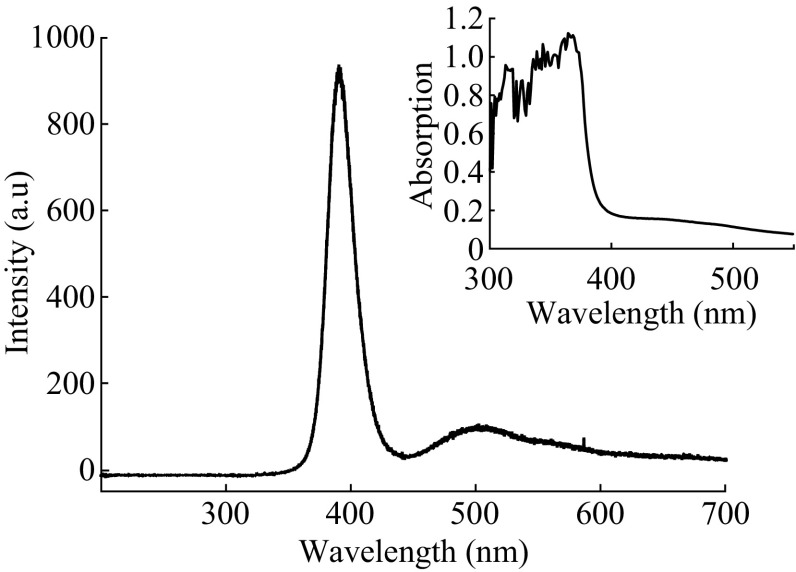
Room temperature photoluminescence spectrum of the as-prepared ZnO nanosheet film. Excitation wavelength: 325 nm. The *inset* picture is the UV–Vis absorption spectrum of the same sample

Figure 4 exhibits the representative room temperature PL spectrum of the ZnO nanosheet films grown in 7.5 mM ZnSO_4_ aqueous solution and annealed at 550 °C for 1 h. A laser with wavelength of 325 nm was used as the excitation source. The strong UV emission peak at about 390 nm could be usually attributed to the free exciton emission from the wide band-gap ZnO [35]. The lower broad peak around 500 nm is usually considered to be the recombination of a photogenerated hole with the single ionized charged state of the defect in ZnO and could be related to the surface oxygen vacancies of the ZnO because the ZnO nanosheet films have much high surface-to-volume ratios [36]. The PL spectrum result indicates that the as-prepared ZnO has few defects [37]. The inset image in Fig. 4 shows the UV–Vis absorption spectrum of this sample, indicating its band gap at nearly 3.25 eV. The band-gap values of ZnO film were calculated by Tauc plot [38, 39].

**Fig. 5:**
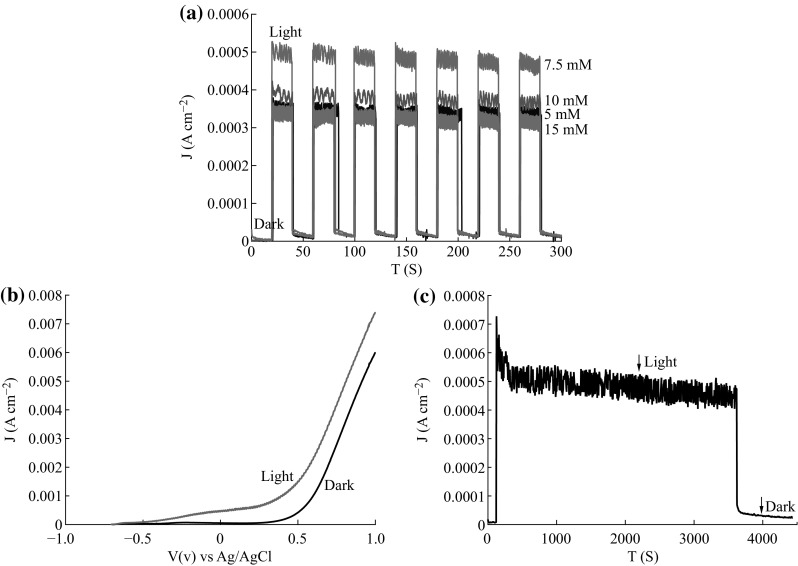
The photoelectrochemical properties of the ZnO nanosheet thin film/ITO electrode. **a** Chronoamperometry measurements at zero bias potential (vs. Ag/AgCl electrode) under chopped light illumination with a light on–off interval of 20 s. **b** LSV curves recorded for the 7.5 mM sample with a scan rate of 10 mV s^−1^ in the applied potentials from −1 to +1 V (vs. Ag/AgCl). **c** The *J* − *T* stability of electrode. All PEC experiments were carried out in a mixture solution of 0.35 M Na_2_S and 0.25 M Na_2_SO_3_ under AM 1.5 G (100 mW cm^−2^)

The photoelectrochemical property of ZnO nanosheet thin film/ITO electrode was measured with an electrochemical workstation. All PEC measurement was carried out in a mixture solution of 0.35 M Na_2_S and 0.25 M Na_2_SO_3_ under AM 1.5 G (100 mW cm^−2^). Figure 5 shows the photoelectrochemical property of ZnO nanosheet thin film/ITO electrode. Its photocurrent was investigated under a bias voltage of 0 V (vs. Ag/AgCl) with a light on–off interval of 20 s, as shown in Fig. 5a. All PEC cell exhibits significant photoresponse under a bias voltage of 0 V (vs. Ag/AgCl), but the sample fabricated at 7.5 mM solution has superior characteristics with a higher photocurrent of 500 μA cm^−2^. The photocurrent density of the photoelectrode varies with the different growth concentrations of ZnSO_4_ aqueous solution, which may be related to the resistance and surface-to-volume ratio of the samples that could affect the light absorption. Linear sweep voltammograms (LSV) curves were recorded for the 7.5 mM sample in the dark and at 100 mW cm^−2^ (AM 1.5) with a scan rate of 10 mV s^−1^ in the applied potentials from −1 to +1 V (vs. Ag/AgCl), as shown in Fig. 5b. The dark scan shows a very small current density in the range of 10 μA cm^−2^, whereas under light illumination a pronounced photocurrent density was observed, implying efficient charge separation and transfer in this nanostructured ZnO. Figure 5c shows the chronoamperometric plots of the ZnO/ITO electrode for about 1 h, which indicated that the photoelectrochemical property was fairly stable under illumination. The photocurrent did not obviously decrease which is very important for the development of practical PEC cells.

**Fig. 6:**
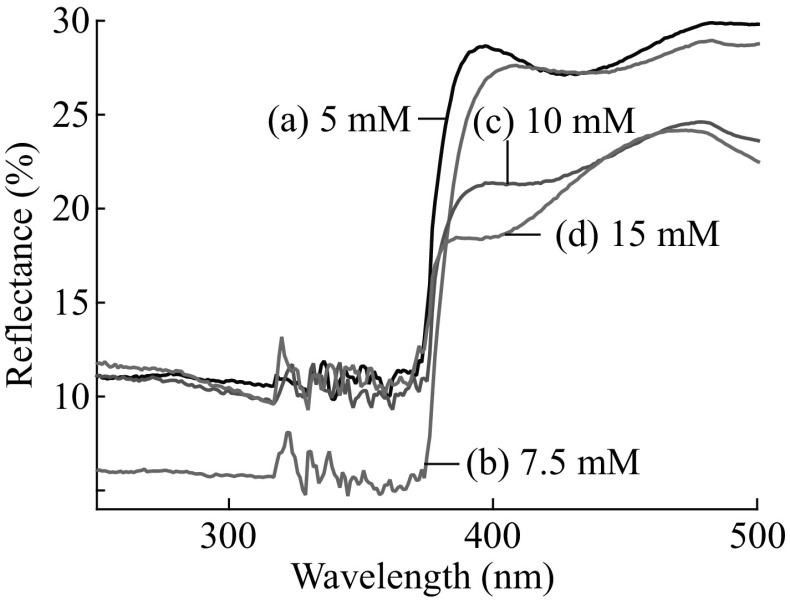
Reflectance spectra of ZnO sheet films on ITO glass grown at different concentrations of ZnSO_4_. *a* 5 mM, *b* 7.5 mM, *c* 10 mM, *d* 15 mM

In order to further understand the photoelectrochemical property of ZnO nanosheet films, the UV–Vis reflectance spectra were also measured by PerkinElmer Lambda 950 UV–Vis spectrophotometer. Figure 6 shows the UV–Vis reflection spectra of the ZnO nanosheet films grown at different concentrations of ZnSO_4_. It can be seen that the 7.5 mM sample had a maximum ultraviolet absorption compared to other samples. The reflectance spectra result was consistent with the photoelectrochemical result. These results clearly demonstrated that the PEC performance strongly depended on the morphology of ZnO and could be optimized through controlling the material growth condition.

## Conclusion

In this paper, we had presented a simple and highly efficient solution-based method to prepare large-scale hexagonal ZnO nanosheet films using a galvanic displacement reaction. The advantages of this solution-processing technique are its simplicity as well as it does not need electric power and supporting agents. The hexagonal ZnO nanosheet films prepared by this method exhibited excellent PEC properties. These results indicated that the ZnO nanosheet film could be applied in low-cost, high-performance photoelectrochemical devices or other application fields.
