# Erratum to: A Facile Self-assembly Synthesis of Hexagonal ZnO Nanosheet Films and Their Photoelectrochemical Properties

**DOI:** 10.1007/s40820-016-0085-5

**Published:** 2016-03-16

**Authors:** Bin Zhang, Faze Wang, Changqing Zhu, Qiang Li, Jingnan Song, Maojun Zheng, Li Ma, Wenzhong Shen

**Affiliations:** 1 Key Laboratory of Artificial Structures and Quantum Control (Ministry of Education), Department of Physics and Astronomy, Shanghai Jiao Tong University, Shanghai, 200240 People’s Republic of China; 2 Collaborative Innovation Center of Advanced Microstructures, Nanjing University, Nanjing, 210093 People’s Republic of China; 3 School of Chemistry & Chemical Technology, Shanghai Jiao Tong University, Shanghai, 200240 People’s Republic of China

## Erratum to: Nano-Micro Lett. DOI 10.1007/s40820-015-0068-y

In the original publication of this paper, Sect. 2.1 the line starting with “… in which the A cell solution is 5–15 mM ZnSO_4_ and B cell solutions is 0.25 M NaOH” is published incorrectly. The corrected line should read as “…in the A cell solution is 0.25 M NaOH and B cell solutions is 5–15 mM ZnSO_4_”.

In Fig. 1, the left solution should be NaOH and the right solution is ZnSO_4_. The corrected figure is provided in this erratum.

**Fig. 1:**
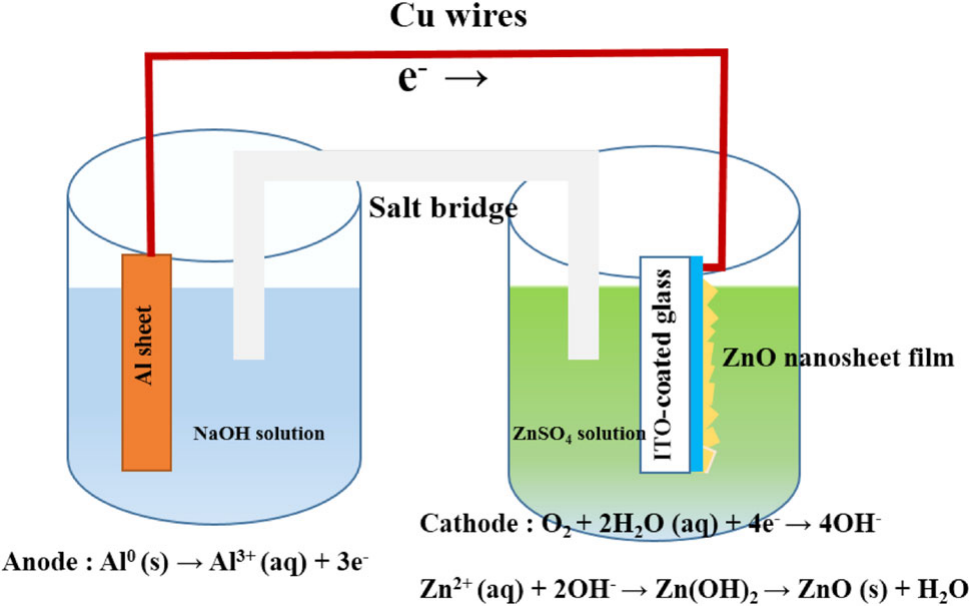
The schematic drawing of the experimental setup used for the fabrication of hexagonal ZnO nanosheet films

